# The complete chloroplast genome sequence of *Rubus sachalinensis* Lévl

**DOI:** 10.1080/23802359.2021.1926354

**Published:** 2021-05-12

**Authors:** Yue Liu, QingYun Song, WenJuan Kang, Fang Yang, Wei Yan

**Affiliations:** a Department of Medical College, Qinghai University, Xining, PR China; b State Key Laboratory of Plateau Ecology and Agriculture, Qinghai University, Xining, PR China

**Keywords:** *Rubus sachalinensis* Lévl, chloroplast genome, phylogenetic analysis

## Abstract

*Rubus* is a medicinal plant distributed in northern China and has high economic and social value. In this study, we reported the complete chloroplast genome sequence of the plant. We determined that the length of the chloroplast genome of *Rubus sachalinensis* Lévl is found to be 155,787 bp and the GC content is 37.24%. The cp genome sequences contains 132 genes, including 37 tRNA genes, eight rRNA genes, and 87 mRNA genes, respectively. The genomic data can help the classification and evolution of Rubus plants, and provide a theoretical basis for the study of *Rubus sachalinensis* Lévl.

*Rubus sachalinensis* Lévl belongs to the genus Rubus in the Rosaceae family. There are more than 900 species worldwide (Ryu et al. 2018), and there are about 194 species in China. They are mainly distributed in forest margins and grasslands at an altitude of 1000–2500 m in the northern region (Editorial Committee of Flora of China, Chinese Academy of Sciences 1985). In Tibetan medicine and Mongolian medicine, the fruit, stems, and leaves of Rubus are the main parts of medicine, and it has a long history of medicinal use. With the in-depth research on Rubus by modern technology, the economic and medicinal value of Rubus has been brought into full play. However, due to the increasing demand for it, in order to provide excellent germplasm resources, it is necessary to pay close attention to the relationship between the species of Rubus, and to detect the chloroplast genome data, which will help the research and protection of the plant. Here, we used genome skimming sequencing data to characterize the complete chloroplast genome sequence of *R. sachalinensis* Lévl.

A modified CTBA method (Doyle 1987) was used to extract total genomic DNA from fresh leaves collected from a plant in Qinghai Province, China (N36°93′31.04″ and E101°69′60.96″). The specimen is deposited in the Herbarium of Qinghai University, the specimen number is 630121200818002LY. In this study, we reported the complete chloroplast genome of *R. sachalinensis* Lévl, sequenced by the Illumina HiSeq2500 platform (Illumina Inc., San Diego, CA), assembled with SPAdes version 3.10.1 (Bankevich et al. 2012), and annotated with CpGAVAS2 (Liu et al. 2012) and Blast search. Phylogenies trees were generated by maximum-likelihood (ML) analysis. Aligned 22 chloroplast genome sequences using MAFFT v7.307 (Katoh and Standley 2013).

Our report shows the complete chloroplast genome sequence of *R.* sachalinensis Lévl (MW085086) is 155,787 bp in length. The typical tetragonal structure consists of a pair of IRs of 51,992 bp, an LSC region of 85,077 bp and an SSC region of 18,718 bp. The GC content of the chloroplast genome is 38.65%. There are 132 predicted genes, 87 protein-coding genes, 37 tRNA genes, and eight rRNA genes.

The ML phylogenetic tree shows that *R.* sachalinensis Lévl is mostly related to *Rubus amabilis* Focke in Rosoideae, with bootstrap support values of 100% (Figure 1), the phylogenetic tree shows that all species of the Rosidae have formed a high-resolution value. In addition, this research lays the foundation for further understanding of the chloroplast genome information of Rubus plants, and provides important information for the development and utilization of characteristic plant resources.

**Figure 1.**
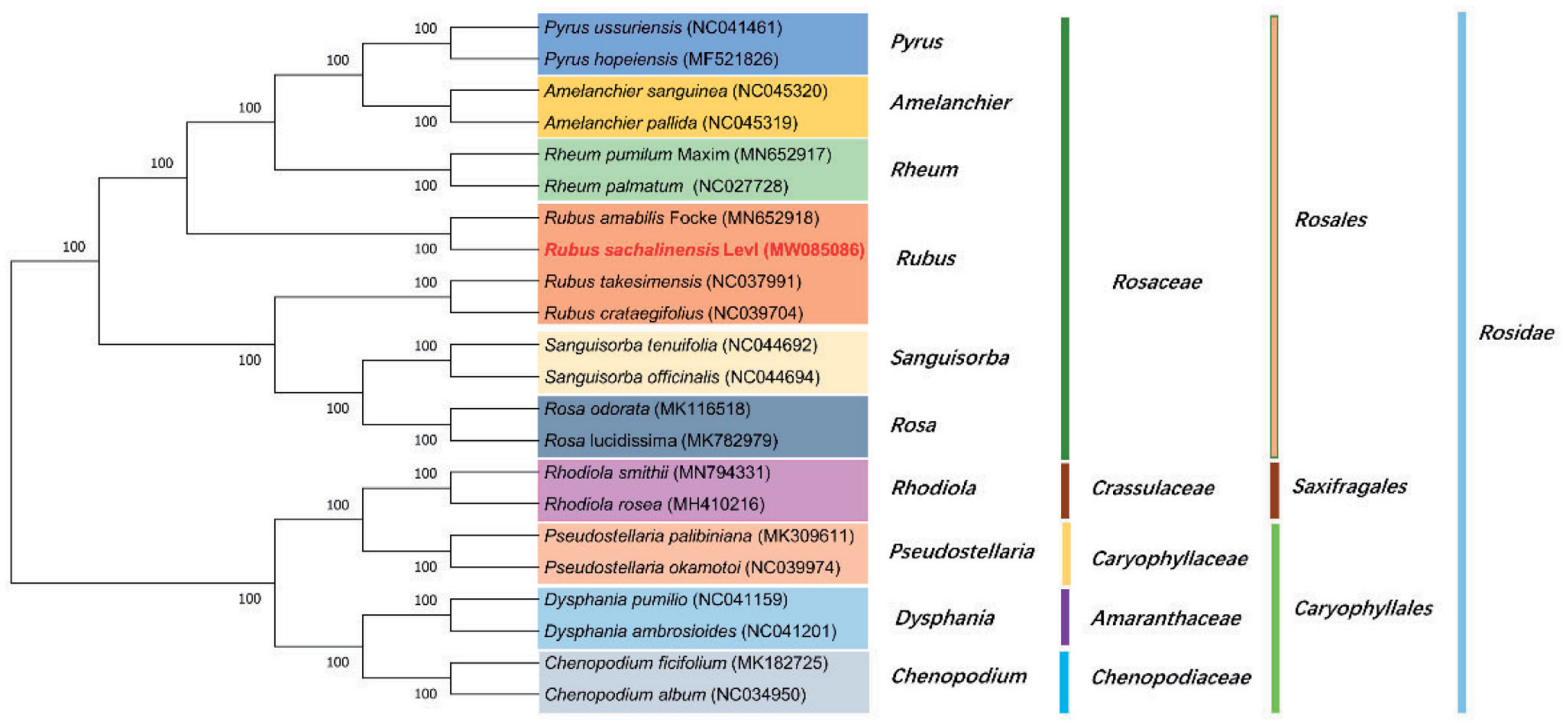
Phylogenetic tree inferred by maximum-likelihood (ML) method based on the complete chloroplast genome of 22 representative species. The *Rubus sachalinensis* Lévl is marked in red and bootstrap values are listed for each branch.

## Data Availability

The data that support the findings of this study are openly available in NCBI GenBank at https://www.ncbi.nlm.nih.gov/nuccore/MW085086.1/, reference number MW085086.
